# COVID-19 Pandemic Experiences Among Adults, Youth, and Childcare Providers: Protocol for a Mixed Methods Study

**DOI:** 10.2196/77521

**Published:** 2025-11-28

**Authors:** Jacinda K Dariotis, Dana A Eldreth, Chunyuan Xi, Iffat Noor, Rebecca Lee Smith

**Affiliations:** 1 Department of Human Development and Family Studies, Family Resiliency Center College of Agricultural, Consumer and Environmental Sciences University of Illinois Urbana-Champaign Urbana, IL United States; 2 Department of Biomedical and Translational Sciences Carle Illinois College of Medicine University of Illinois Urbana-Champaign Urbana, IL United States; 3 Department of Health and Kinesiology College of Allied Health Sciences University of Illinois Urbana-Champaign Urbana, IL United States; 4 Department of Pathobiology College of Veterinary Medicine University of Illinois Urbana-Champaign Urbana, IL United States; 5 Carl R. Woese Institute for Genomic Biology Carle Illinois College of Medicine University of Illinois Urbana-Champaign Urbana, IL United States

**Keywords:** COVID-19, pandemic, public health, vaccine hesitancy, mixed methods, family, coping, youth

## Abstract

**Background:**

The COVID-19 pandemic challenged families, youth, and frontline workers, including childcare providers. Studying lived experiences before, during, and near the pandemic’s end from multiple perspectives provides a more holistic and deeper understanding of its effects and impacts.

**Objective:**

This study investigated how parental, childcare provider, and youth stress, mental health, and role overload relate to individual coping and family functioning, as well as vaccine attitudes and uptake patterns among youth, parents, and childcare providers. Information learned from this investigation will inform policy and messaging for future public health crises.

**Methods:**

This study is an explanatory sequential mixed methods study designed to capture the voices of parents of children younger than 18 years of age, childcare providers, and youth aged 12-17 years through surveys and interviews. This retrospective cross-sectional study began with a web-based survey that included demographic questions and validated scales to assess personal well-being, household and family dynamics, behavioral problems, and vaccination-related perceptions, attitudes, and behaviors. Open-ended responses about pandemic experiences for themselves and their families were included. A subsample of parents, youth, and childcare providers was selected for in-depth interviews about their pandemic-related experiences. Descriptive statistics were used to summarize demographic characteristics, and internal consistency was assessed for all survey measures using Cronbach α. Future studies will use inferential statistical techniques to analyze survey measures, thematic analysis for open-ended survey responses and interview data, and mixed methods data integration to synthesize quantitative and qualitative findings.

**Results:**

Data collection for the study began in August 2022 and finished in August 2023. Data analysis is currently in progress to address research questions, and study preparation and dissemination efforts are underway. A total of 506 adults and 93 youths answered a study survey, and 45 adults and 21 youths completed in-depth interviews. Among the 506 adults, 166 were childcare providers. The adult sample had a mean age of 42.8 (SD 9.15) years and was predominantly female (467/506, 92.3%), with 9.7% (49/506) identifying as Black, 4.7% (24/506) as Hispanic, and 81.2% (411/506) being parents of children aged 17 years or younger. The youth sample had a mean age of 14.5 (SD 1.63) years, and 55.9% (52/93) were female, 6.4% (6/93) were Black, and 17.2% (16/93) were Hispanic. Several dyads and triads participated. The sample included 42 parent-child dyads, 3 parent-parent dyads, 2 parent-parent-child triads, and 21 parent-child-child triads.

**Conclusions:**

These data will be used to understand the diverse experiences of families, youth, and childcare providers during and after the COVID-19 pandemic. This includes successful and unsuccessful adaptations, responses to policies and mandates, and the unmet needs for health messaging, programs, policies, and services. This research aims to guide the development of effective policies and public health communication, fostering scalable and sustainable messaging resources.

**International Registered Report Identifier (IRRID):**

DERR1-10.2196/77521

## Introduction

### Background

The COVID-19 pandemic significantly impacted families, youth, and frontline workers, including childcare providers. Much of the impact resulted from isolation, social distancing mandates, school closures, loss of loved ones, and economic hardships. For some families, these factors resulted in role reversal for youth who assumed adult-like responsibilities. Public health adherence also dwindled as mixed messaging increased vaccine hesitancy in adults, including parents of youth [1]. Despite the many challenges, silver linings and elements of resilience emerged. To prepare and respond to future public health crises effectively, adopting a systems perspective is essential for illuminating the complex, interwoven lived experiences of families, youth, and childcare providers.

The pandemic affected children’s mental health and well-being, with dyadic studies highlighting the importance of family relationships on mental health. A longitudinal study of mothers and their adolescent children (prepandemic in September 2019 and follow-up in May-June 2020) reported increased depression and decreased anxiety, with depressive symptoms being most significant in adolescents [2]. The authors suggest findings reflect resiliency (eg, more family time) and risk factors (eg, financial and interpersonal hardships) early in the pandemic. Other dyadic studies discovered that increased family activities helped mitigate the effects of reduced peer interactions during the pandemic [3]. Actor-partner models revealed that adolescents’ perceived social support was linked to lower parental anxiety, though the reverse was insignificant [4]. Additionally, the relationship between perceived social support and anxiety in parents and their adolescents was mediated by perceived family resilience. These findings highlight the need to further explore other aspects of family dynamics that foster resiliency.

Households with children were more likely to experience job loss and reduced income [5]. Moreover, parents serving as frontline and essential workers worked longer hours under greater stress than prepandemic, leaving children to care for their own and siblings’ instrumental and emotional needs and parents’ needs [6]. These circumstances disproportionately impacted youth, especially children forced to shoulder adult responsibilities to adapt to pandemic demands. Role reversal during COVID-19 has focused on adult youth, suggesting that the role shifts may negatively affect their relationship with parents and contribute to poorer mental health outcomes, including stress, anxiety, and depression [7,8]. Given the changes in family dynamics and impact on youth, it is important to understand how these added stressors at home impacted them during the pandemic.

Another group overwhelmingly impacted by the pandemic was childcare providers. As essential workers, childcare providers returned to work while other parents worked from home or stayed at home. Extensive research has investigated how the pandemic exacerbated childcare providers’ pre-existing financial and job-related stress [9,10]. Given their circumstances, the youth of childcare providers were more vulnerable to pandemic-related economic hardships and increased pressures for family adaptations. To our knowledge, no research has been conducted to determine the impact of the pandemic on the youth and families of childcare providers. Exploring this in this study will enable policymakers to address the unique impacts of the pandemic on childcare providers who are critical to child development and the labor force.

Families were also impacted by mixed messaging on how to protect oneself and others from COVID-19 [11]. Although vaccination is one of the most effective strategies to promote public health safety, vaccination rates have been declining [12]. A case study of three participants with differing vaccination views revealed shared and contrasting attitudes shaped by family health priorities, past trauma, and distrust in media and politicians [13]. Recommendations emphasized respecting autonomy and offering clear information to support informed decisions [13]. Exploring the characteristics and rationale of those who do and do not adhere to public health recommendations will enable us to better understand factors underlying decision-making and potential areas for improved public health messaging to reach everyone.

The pandemic had both positive (silver linings) and negative (thorns) influences on mental health, daily functioning, behavioral health choices, and long-term outcomes [14,15]. Silver linings encompassed personal growth, increased time with family and friends, and greater appreciation and gratitude [16]. Thorns included the death of loved ones, reduced medical services, chaos replacing routines, unemployment, food insecurity, isolation and lost social support, and educational interruptions [17]. Our qualitative study of parents and youth between the ages of 12-17 years found similar thorns and silver linings [18], as well as parents expressing concerns about developmental delays due to mandates such as masking and social isolation. Despite this, signs of resiliency emerged related to maturation and cultivating adaptive coping strategies such as finding ways to socialize safely, prioritizing health, and finding new leisure activities. Further understanding successful adaptations made during the COVID-19 pandemic will elucidate factors promoting resiliency during public health crises. These effective adaptations may inform policies designed to enhance the resiliency of families.

### Aims and Objectives

Taking a systems approach, this study innovatively collected survey and interview data about multiple children in families and from multiple family members (dyads and triads) and childcare providers. By using an explanatory sequential mixed methods design [19], this study provides an evidence base for how families, youth, and childcare providers have and continue to cope with and adjust to the COVID-19 pandemic. The two primary objectives of this study are: (1) to investigate how parental, childcare provider, and youth stress, mental health, and role overload relate to individual coping and family functioning. Parent-youth dyads and parent-youth-youth triad data will uncover patterns in shared and nonshared experiences and differential needs to inform prevention and intervention strategies (eg, policies, programs, and practices); and (2) to predict patterns of vaccine attitudes and uptake in youth, parents, and childcare providers. We can explore parent and childcare provider vaccine attitudes and uptake preferences for youth.

## Methods

### Setting

This study was conducted in the United States, with most participants recruited from the Midwest region.

### Study Population and Recruitment

Participant inclusion criteria included parents or caregivers aged 18 years or older caring for youth. Youth participants between the ages of 12 and 17 years old at the time of the survey could participate with parent consent. Childcare providers aged 18 years or older could participate regardless of whether they had minor children. All participants had to be proficient in written and spoken English and located in the United States.

Recruitment and enrollment efforts occurred between August 2022 and August 2023 (with one final youth participant enrolled in March 2024). Participants were recruited via an existing study at the university, local schools, community venues serving low-income families, listservs, newsletters, and community events (eg, disability expo). Recruitment occurred via flyers and emails with a study overview and study team email. Participants were encouraged to email the study team to receive a web-based link to the study eligibility screener. Efforts to diversify the sample included attending in-person community events tailored to select populations (eg, resource expo for families of children with developmental disabilities); community organizations serving families and youth; social media postings for local school districts and public health departments; and advertisements on family listservs, childcare resources, and statewide childcare provider listservs, and university faculty and staff newsletters.

Interviewees were selected among survey participants using purposive sampling based on characteristics essential to the study. A phased selection process was used to maximize sample diversity regarding demographics, vaccination status, and views. The eligibility and screening criteria for each phase are shown in Table 1. Selected participants included those who fully or partially completed the survey. Phase 1 recruitment focused on family dyads (parent-youth) and triads (parent-youth siblings) and childcare providers to meet study objectives. During weekly meetings throughout interview data collection, the study team discussed emergent themes from interviews and whether we were reaching saturation [20] or recruitment needed to be modified to obtain a more diverse sample. Phase 2 recruitment prioritized participants who were self-identified as Black or Hispanic, childcare providers, of lower socioeconomic status, unvaccinated, or had less favorable attitudes toward vaccines. Phase 3 recruitment centered on participants with a score of 1.5 SD above the mean on survey ratings of measures demonstrating hardship associated with the pandemic (eg, household chaos and stress) or nonmainstream beliefs (eg, vaccine hesitancy). Phase 4 recruitment focused on increasing youth representation. Recruitment occurred via email, followed by phone calls to parents.

**Table 1:** Eligibility criteria and priority characteristics for interviewee recruitment.

Phase	Eligibility criteria	Priority characteristics
1	Families with children younger than 18 years of age Childcare providers	Dyads (parent-youth and triads (parent-youth siblings)
2	Adults, youth, and childcare providers meeting any of the following criteria: Racially diverse Lower socioeconomic status relative to the sample Vaccination status and intentions	Self-identified as Black or Hispanic Annual household income less than US $49,000 Unvaccinated No intention to vaccinate the child
3	Adults meeting any of the following criteria: Outliers in survey responses related to the impacts of the pandemic and attitudes about vaccines Nonmainstream beliefs about vaccines	1.5 SD above the mean on household chaos, perceived stress, and perceived economic hardship 1.5 SD below the mean for trust in health care providers’ recommendations on vaccines for children, and trust in public health agencies Open-ended responses suggesting nonmainstream beliefs about vaccines or the pandemic (eg, negative vaccine attitudes and conspiracy theories)
4	Youth-targeted recruitment	All youth who partially or fully completed the survey

### Study Design and Procedures

**Figure 1:**
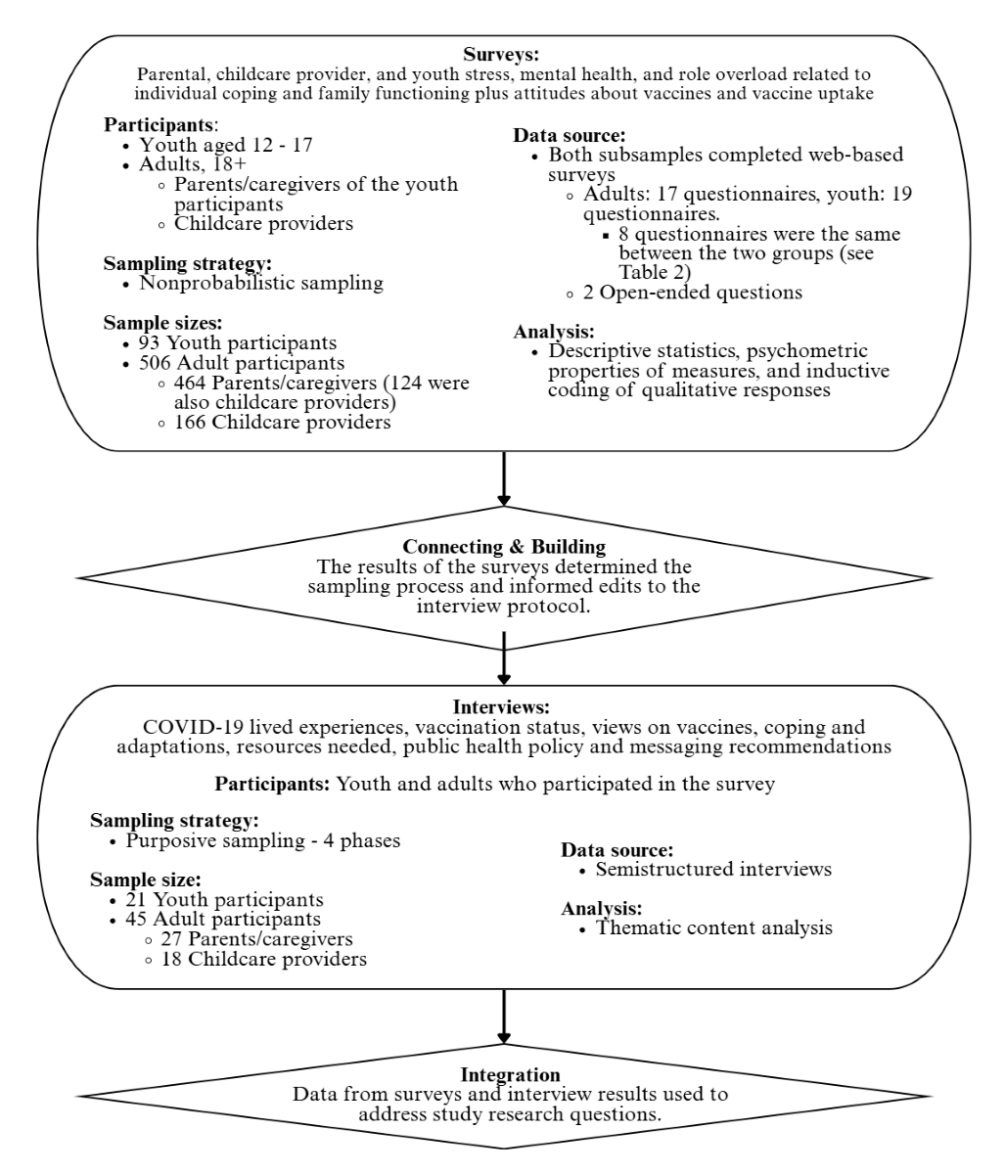
Procedural diagram.

This study was an explanatory sequential mixed methods study [21] designed to capture the voices of parents of children younger than 18 years of age, youth aged 12-17 years, and childcare providers through surveys and interviews. This study began with a web-based survey that included demographic questions, standardized and validated scales to assess constructs of interest, and open-ended responses about pandemic experiences. Based on survey responses and their willingness to participate in a follow-up data collection, a subsample of youth and adult participants was invited to be interviewed about their experiences during the pandemic. The procedural design is shown in Figure 1; analysis of qualitative data and integration of data sources are not addressed in this paper.

Adult and youth surveys were administered using the web-based survey platform Qualtrics (Qualtrics, Provo, UT). Adults who met the eligibility criteria via the survey screener received a personalized link to the main survey. Parents of children between the ages of 12 and 17 years had the opportunity to provide consent for their children to participate in the study. Consented youth aged 12-17 years received a link to the main survey, where they provided assent prior to the initiation of their web-based survey. The survey was designed to take approximately 40 minutes for adults and 20 minutes for youth. To maximize survey completion, reminders were programmed into Qualtrics to automatically send an email reminder to complete the survey every 3 days.

**Figure 2:**
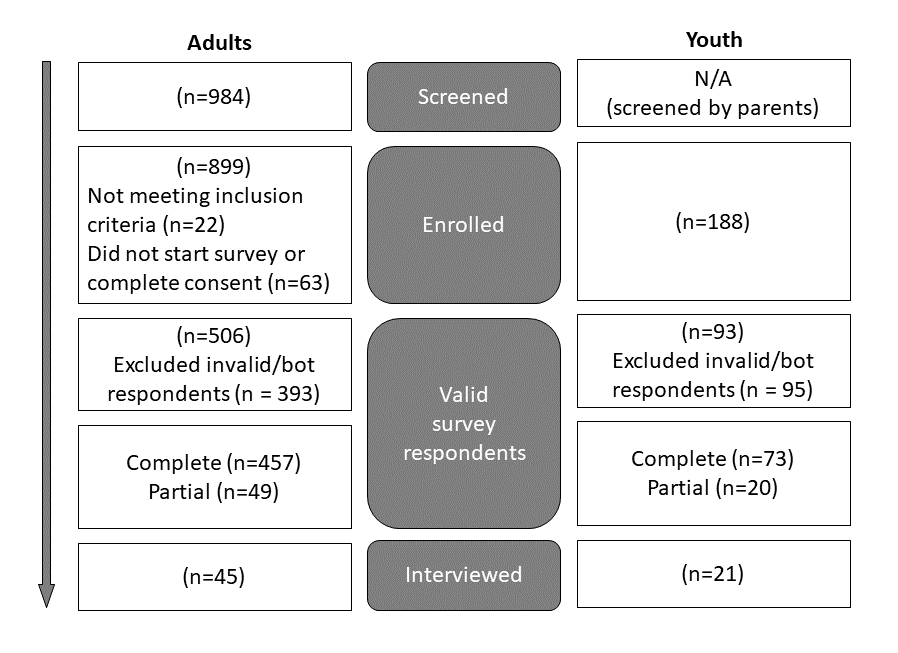
Study flow diagram.

Initially, a screener survey with anonymous links was shared through university listservs and a university-based preschool, and these links could be shared with others. For the other listservs, individuals interested in participating were required to email the study team to learn more about the research study and receive a personalized link to the screener survey through Qualtrics. To ensure participants were valid respondents and to guard against bots, we incorporated a validation protocol consisting of numerous automated and manual checks that are presented in Textbox 1. Due to the rollout of the surveys through different listservs, data validation checks were progressively added in three phases. During each phase, the study team discussed cases not meeting these criteria to determine if they should be excluded from analyses. Figure 2 shows the number of participants identified as invalid respondents.

**Data validation protocol.**

**Phase 1: Participants accessed the screener via an anonymous link or emailed the study for a personalized link**

- Step 1 (automated within Qualtrics)
  Common to both the screener and the main survey
  Completely automated public turning (CAPTCHA)
  Logic that rejected multiple respondents entering the same email addresses
  Prevention of indexing to stop search engines from showing the link to the survey in search results
  Screener survey Viewing an embedded video with a question, “Please type the word that this video is about.”
  Specific to main survey
  Viewing an embedded picture with a multiple-choice question, “Where does this character live?”
  2 midsurvey checks: Check 1: solve this equation 1+2=3; check 2: “how seriously have you taken the survey?”
- Step 2 (manual inspection)
  Screener and main survey
  Reviewed respondents’ answers to embedded questions
  Inspected duplicate IP addresses to ensure they originated from families only
  Reviewed open-ended duplicate or nonsensical open-ended responses
  Checked for unusual email addresses (example123456@gmail.com)
  Cross-reference discrepancies in screener versus main survey (date of birth)
  Removed participants who completed the survey too quickly (<15 min for adults and <7 min for youth)
  Removed participants with duplicate start dates by the minute
  Reviewed duplicate or nonsensical open-ended question responses
  Called respondents who did not meet these criteria to determine validity
  Discuss suspicious cases during study meetings to exclude from analyses

**Phase 2: Participants were required to email the study for a personalized link to the screener and answer screening questions**

- Steps 1-2 repeated from Phase 1
- Step 3 (manual inspection)
  Email review for odd grammar, repeated text, and emails received in quick succession
  Checked to make sure screener information matched what was provided in the email

**Phase 3: Programmed additional checks into Qualtrics and called participants to ensure valid respondents**

- Steps 1-3 repeated from phases 1 and 2
  Validation check
    - Added questions about city, state, and zip code to confirm with the IP address

A subset of survey participants was invited to complete interviews based on criteria described previously. These participants were emailed or called to determine their interest in being interviewed and their schedule to set up a time. Parents of children aged 12-17 years were asked if their children would be willing to be interviewed, and if so, when would be the best time to speak with them. Some parents helped with scheduling their children’s interviews. Interviews were conducted via Zoom (Zoom Video Communications Inc), telephone, or in-person based on participant preference and distance.

### Measures

#### Overview

Standardized and validated questionnaires were programmed in Qualtrics. The web-based survey was pilot tested by the study team and Center staff to ensure usability, readability of questions, and technical functionality. Data were also reviewed to ensure proper data collection (ie, branching logic, forced choice questions, values within ranges, and completion time). Modifications were programmed into Qualtrics based on feedback and data quality until the survey was ready to publish on the web for public use.

Two surveys were administered: one for adults and one for youth aged 12-17 years. Survey measures, including respondent type and timepoint, are presented in Table 1. The adult web-based survey contained 17 standardized questionnaires, demographic questions, and two open-ended questions. Parents also answered demographic questions about all their children (eg, age, gender, and school openness status) and then a series of questions about their two oldest children, aged 17 years and younger. This series included questions about parentification, behavioral strengths and difficulties, confidence about having the child vaccinated, and open-ended questions related to the impact of the pandemic on each child. All participants completed questionnaires in a fixed order. To maximize readability, only one questionnaire was presented on each page. Matrix-style survey questionnaires with Likert scale radio buttons had repeating headers for clarity. Questionnaires requiring participants to compare various timepoints were presented side by side with drop-down Likert scale options. All survey items were forced responses, except for income-related household demographics. Other questionnaires that were not forced responses are indicated in Table 2. If participants did not feel comfortable providing these responses, we did not want that to prohibit them from completing the survey. Participants could not go back to previous surveys to modify their responses.

**Table 2:** Survey measures by respondents and timepoints.

		Adults	Youth	Timepoint
**General characteristics**				
	Demographics, employment, earnings^a^, household	✓	✓	Post
	School-related		✓	Post, mid
	Information about their children	✓		post
	Childcare provider information^b^			Post, mid
**Personal well-being and beliefs**				
	Strengths and Difficulties Questionnaire^c^	✓	✓	Post, pre
	Confusion, Hubbub, and Order Scale	✓	✓	Post, mid, pre
	Perceived Economic Hardship^d^^,^^e^	✓	✓	Post, mid, pre
	Perceived Stress Scale	✓	✓	Post
	Personal Values	✓	✓	Post
	Parentification Inventory^c^	✓	✓	Post, pre
	Media Attention	✓	✓	Post
	Depression Anxiety Stress Scale	✓		Post
	Hong Psychological Reactance Scale	✓		Post
	Conspiracy Mentality	✓		Post
	Cultural Attitudes	✓		Post
	Religiosity and Ideology	✓		Post
	PROMIS^f^—Emotional-Behavioral Dyscontrol		✓	Post
	PROMIS—Cognitive Functioning		✓	Post
	PROMIS—Sleep-Related Disturbance		✓	Post
	PROMIS—Emotional Distress Anxiety Pediatric Item		✓	Post
	PROMIS—Depression		✓	Post
	PROMIS—Positive Affect and Well-being		✓	Post
	Child Adolescent Mindfulness Measure		✓	Post
	Emotional Regulation Questionnaire		✓	Post
	Brief COPE^g^		✓	Post
	Adverse Childhood Experiences Checklist^a^		✓	Ever
	Social Competence Scale		✓	Post
**Vaccine perceptions, attitudes, and behaviors**				
	Parent Attitudes about Childhood Vaccines	✓		Post, pre
	Vaccine Risk Analysis	✓		Post
	Vaccine Confidence Index^c^	✓		Post, pre
	Vaccine Hesitancy Scale	✓		Post, pre
	Extended Parallel Process Model	✓	✓	Post

^a^Not forced response.

^b^Information only asked about childcare providers.

^c^Parents answered about the two oldest children, aged 17 years and younger.

^d^Youth only reported one timepoint.

^e^Partial forced response.

^f^PROMIS: Patient-Reported Outcomes Measurement Information System.

^g^COPE: Coping Orientation to Problems Experienced.

Childcare providers also answered questions related to their roles as providers and an open-ended question related to the impact of COVID-19 on them as childcare providers. The youth web-based survey contained 19 questionnaires, some overlapping with adults and others unique to youth (eg, mental health), including two open-ended questions. Survey measure descriptions are organized by those common to both adult and youth surveys, followed by measures unique to adults and then unique to youth. Published scoring guidelines were used for all standard scales and questionnaires. Time points are assigned as pre (prior to March 2020), mid (during the pandemic—March 2021), and post (at the time of data collection, near the end of the pandemic—2022-2023).

#### Measures Common to Adult and Youth Surveys

The Strengths and Difficulties Questionnaire [22] was used to assess the youth’s behavioral problems and prosocial behaviors from both parent and youth perspectives. This 25-item measure includes five subscales: Hyperactivity, Emotional Symptoms, Conduct Problems, Peer Problems, and Prosocial Behavior. Ratings were provided using a 3-point Likert scale (0=not true, 1=somewhat true, and 2=certainly true). Five items were reverse-coded so that higher scores denote more of that characteristic. Subscale scores are calculated by summing their respective items, and the Total Difficulties score is calculated by summing the scores of all four problem scales.

Household environmental and family chaos were evaluated using the Confusion, Hubbub, and Order Scale [23]*,* a 15-item measure rated on a 4-point Likert scale from 1=very much to 4=not at all. Eight items are reverse-coded. Scores are calculated by summing all items. Higher scores indicate greater levels of disorganization, confusion, and noise within the home environment.

The Perceived Economic Hardship Questionnaire [24] was used to assess economic hardship. This 17-item measure comprises four subscales: (1) Not Enough Money for Necessities (6 items, rated from 1=strongly agree to 5=strongly disagree); (2) Inability to Make Ends Meet (2 items, with one item rated from 1=no difficulty at all to 5=a great deal of difficulty, and another reverse-coded item rated from 1=very short of money to 5=more than enough money left); (3) Financial Strain (2 items rated from 1=almost never to 5=almost always); and (4) Economic Adjustments (7 items rated from 1=not at all to 5=extremely). Scores are calculated by averaging all the items in each subscale. Higher mean scores indicate greater perceived economic hardship. Adults provided ratings for the Not Enough Money for Necessities and Inability to Make Ends Meet subscales at all three time points, and Financial Strain and Economic Adjustments were assessed only at the postpandemic time point. Youth provided ratings for the Financial Strain subscale only. Financial Strain and Economic Adjustment scales were not forced responses.

Perceived stress was assessed using the Perceived Stress Scale [25]*,* a 10-item measure evaluating how stressful individuals found the specific situations over the past month. Both adults and youth rated items on a 5-point Likert scale from 0=never to 4=very often. Four items are reverse-coded, and the total score is calculated by summing all 10 items.

Personal values were evaluated with the Personal Values Scale [26]*,* an 8-item measure. This scale assesses the significance individuals attribute to various aspects of their lives, including physical health, mental well-being, and interpersonal relationships. Adults and youth rate items on a 10-point Likert scale from 1=least important to 10=most important. Total scores are calculated by summing all eight items, with higher scores reflecting stronger personal values.

Parentification was assessed using the Parentification Inventory [27], which evaluates the extent to which youth engaged in adult-like roles and responsibilities typically reserved for parents. This 22-item measure includes three subscales: Parent-Focused Parentification, Sibling-Focused Parentification, and Perceived Benefits of Parentification. Responses are recorded using a 5-point Likert scale ranging from 1=never true to 5=always true. Subscale scores are computed by averaging corresponding items.

Attention to media was assessed using the Media Attention Survey [28], which measures individuals’ level of attention to news stories across health-related topics, including health and nutrition, COVID-19, and other issues. Participants were asked how much attention they pay to these topics when reading newspapers, watching television news, or going on the internet. The survey includes three items, each rated on a 5-point Likert scale ranging from 1=none at all to 5=a great deal. Scores are computed by averaging the three items, with higher scores representing greater attention to media.

The Extended Parallel Process Model [29] was included to assess perception of COVID-19 vaccine efficacy using the framework for effective communication of health-related information. This 15-item scale is rated on a 5-point Likert scale ranging from 1=strongly disagree to 5=strongly agree. Mean scores were calculated for items within the Severity Subdomain, Susceptibility Subdomain, and mean for all items. Higher scores represented greater fear of susceptibility and severity from COVID-19. For the Response Efficacy Subdomain, which measures beliefs about the effectiveness of the COVID-19 and flu vaccines, mean scores were calculated, with higher scores indicating greater perceived efficacy.

#### Measures Unique to the Adult Survey on Well-Being and Beliefs

General psychological distress, including symptoms of depression and anxiety, was assessed using the Depression Anxiety Stress Scale [30]*.* This 21-item measure includes three subscales: Depression, Anxiety, and Stress. Adults rate items on a 4-point Likert scale ranging from 0=not at all to 3=very much or most of the time. Subscale scores are computed by summing the corresponding items and multiplying the total by 2 to match the 42-item version [30]. Higher scores indicate greater depression, anxiety, and stress.

Psychological reactance was measured using the Hong Psychological Reactance Scale [31], an 18-item measure with four subscales: Freedom, Conformity Reactance, Behavioral Freedom, and Reactance to Advice and Recommendations. Adults report about their own beliefs on a 5-point Likert scale from 1=strongly disagree to 5=strongly agree, with higher scores indicating greater psychological reactance. Scores are calculated by averaging all the items in each subscale.

Conspiracy mentality was assessed using the 5-item Conspiracy Mentality Questionnaire [32], which assesses individuals’ general tendency to endorse conspiratorial beliefs. Adults rated their agreement with each item (eg, “Government agencies closely monitor all citizens”) on a 5-point Likert scale from 1=certainly untrue to 5=certainly true, with higher scores indicating greater conspiracy thinking. A total score was calculated by averaging all items.

Cultural attitudes were measured using a 12-item scale Cultural Attitudes Questionnaire [33], which assesses individuals’ cultural value orientations across four domains: hierarchy, egalitarianism, fatalism, and individualism. Adults rated how strongly they agreed with each statement (eg, “Society works best if power is shared equally”) on a 7-point Likert scale ranging from 1=strongly disagree to 7=strongly agree, with higher scores indicating stronger endorsement of that cultural orientation. Each subscale score was calculated by averaging three corresponding items.

Religiosity and political ideology, and affiliation were measured using the 4-item Religiosity and Ideology questionnaire [34]. Religiosity was evaluated using one item on a 5-point Likert scale ranging from 1=very little to 5=a great deal. Higher scores for religiosity represent a greater role of religion in everyday life. Political ideology was measured using two items on a 5-point Likert scale, representing 1=very liberal to 5=very conservative. These items were averaged with higher scores representing more conservative views. Political affiliation is identified as Democrat, Republican, Independent, No preference, and Other party*.*

#### Measures Unique to the Youth Survey

Specific mental, physical, and social health outcomes were assessed using several Patient-Reported Outcomes Measurement Information System surveys: Emotional-Behavioral Dyscontrol [35] is an 8-item measure that evaluates emotional regulation and behavioral control. Responses are recorded using a 5-point Likert scale from 1=never to 5=always. The Emotional-Behavioral Dyscontrol total score is calculated by summing all items, with a higher score indicating greater emotional and behavioral dyscontrol.

Cognitive Functioning [36] is an 8-item measure used to assess cognitive performance. The first four items assess the frequency with which participants encounter specific cognitive challenges, using a 5-point Likert scale ranging from 1=never to 5=very often. The remaining four items evaluate participants’ current levels of difficulty performing cognitive tasks, also rated on a 5-point Likert scale ranging from 1=none to 5=cannot do. All items are reverse-coded, and the total score is calculated by summing the responses to all items. Higher scores indicate better perceived cognitive functioning.

Sleep-Related Disturbance [37] is a 6-item measure evaluating sleep quality and disturbances. The first item assessed general sleep quality, ranging from 1=very poor to 5=very good. The remaining five items rated sleep disturbance behaviors on a different 5-point Likert scale from 1=not at all to 5=very much. One of these five behavior items is reverse-scored, and the total score is calculated by summing across the five items. Higher scores indicate greater severity of sleep disturbance.

Emotional Distress-Anxiety [38] is an 8-item scale measuring anxiety and emotional distress in youth. Youth are asked to rate how frequently they experienced the described feelings or situations during the past 7 days. Responses are recorded on a 5-point Likert scale ranging from 1=never to 5=almost always. The total score is calculated by summing scores for all items, with a higher score indicating a greater level of pediatric emotional distress and anxiety.

Depressive Symptoms [38] were measured using 8 items rated on a 5-point Likert scale ranging from 1=never to 5=almost always. The total weighted score was calculated by summing the raw scores of all items, multiplying the result by the number of items, and then dividing it by the number of items completed. A higher score indicates greater severity of depressive symptoms.

The Positive Affect and Well-Being [35] was used to measure positive affect and well-being. This is a 9-item measure rated on a 5-point Likert scale ranging from 1=never to 5=almost always. The total score is calculated by summing all items. A higher score indicates a greater sense of positive affect and well-being.

Mindfulnes*s* was measured using the Child and Adolescent Mindfulness Measure [39]*,* a 10-item scale rated on a 5-point Likert scale from 0=never to 4=always true. All items are reverse-scored, and the total score is computed by summing all items. Higher scores reflect greater mindfulness.

Emotional regulation skills were assessed using the Emotion Regulation Questionnaire [40], a 10-item measure comprising two subscales: Cognitive Reappraisal (6 items) and Emotion Suppression (4 items). Subscale scores are calculated as the mean of their corresponding items. Items are rated on a 7-point Likert scale ranging from 1=strongly disagree to 7=strongly agree. Higher scores on the cognitive reappraisal subscale indicate greater effectiveness in reducing the experiential and behavioral components of negative emotions, whereas higher scores on the emotion suppression reflect a less effective strategy for reducing negative emotions.

Coping strategies for stressful events were measured using the Brief Coping Orientation to Problems Experienced [41]*.* This 28-item instrument assesses 14 coping strategies with 2 items in each: Self-Distraction, Instrumental Support, Active Coping, Behavioral Disengagement, Denial, Venting, Substance Use, Humor, Emotional Support, Positive Reframing, Planning, Acceptance, Religion, and Self-Blame. Responses were recorded using a 4-point Likert scale ranging from 1=do not do this at all to 4=usually do this a lot. All items were reverse-coded, and subscale scores were computed by summing the responses to the corresponding items. Higher scores indicate better coping.

The Adverse Childhood Experiences Questionnaire [42] is a 10-item measure used to identify traumatic events experienced during childhood, specifically involving abuse, neglect, and household dysfunction. Each item prompts respondents to indicate whether specific events occurred in their child’s life, with answers scored as 1=yes and 0=no. A total Adverse Childhood Experiences Questionnaire score is generated by summing the responses across all items. For this study, only eight nonreportable items were used. Scores range from 0 to 8, with a higher score indicating a greater exposure to adverse childhood experiences.

The National Survey of Children’s Health Social Competence Scale is a 9-item survey used to measure social competence in adolescents, defined as the positive skills needed to interact respectfully, work well in groups, follow social norms, and resolve conflicts effectively [43]. Three items are rated on a 5-point Likert scale ranging from 0=not at all like me to 4=exactly like me, and 6 items rated on a 5-point Likert scale (0=none of the time to 4=all of the time). The sum is calculated with higher scores denoting greater social competence.

#### Measures Unique to the Adult Survey on Vaccination

Parent Attitudes about Childhood Vaccines [44] was used to measure parents’ beliefs about childhood vaccinations based on their behavior, views on safety and efficacy, general attitudes, and hesitancy. Nine of the 12 items from the original scale were used in this study. Two items were dichotomous (yes or no), and one item was trichotomous (yes or no or don’t know). The other items were rated on the Likert scales as follows: two items ranging from 0=do not trust at all to 10=completely trust; two items ranging from 1=not at all concerned to 4=very concerned; one item ranging from 1=not at all hesitant to 4=very hesitant. Mean scores of three items were calculated for the Self-efficacy subscale and two items for the General Attitudes subscale. Hesitancy was represented by the response to “Overall, how hesitant about childhood shorts would you consider yourself to be?” Items in the Behavioral subscale were summed and then categorized into tertiles, representing varying levels of hesitancy from low to high. Higher scores on all subscales indicate more negative attitudes about childhood vaccines.

Vaccine Risk Analysis [44] is a 5-item measure used to measure thoughts on benefits and risks regarding vaccinating themselves and their children. Ratings are on a 10-point Likert scale for two items, where 0=not at all beneficial to 10=extremely beneficial, two items rated 0=no risk to 10=extreme risk, and one item rated 1=risks far outweigh benefits to 7=benefits far outweigh risks. The composite vaccine benefit-risk index is computed by taking the mean of the 5 items, with 3 items reverse-coded, and item 5 rescaled to match the other 4 items. The mean of all 5 items is computed, with higher scores representing greater perceived risks from vaccines.

Vaccine Confidence Inventory [45] is a 4-item scale used to assess how confident participants are about the COVID-19 and flu vaccine. Items were rated on a 7-point Likert scale ranging from 1=strongly disagree to 7=strongly agree*.* This study omitted the question “vaccines are compatible with my religious beliefs.” Mean scores were calculated for the items related to COVID-19 vaccines and flu vaccines. Higher scores represent greater confidence in these vaccines.

A modified version of the Vaccine Hesitancy Scale [46] was used to assess overall vaccine hesitancy using 15 items. Items are rated on a 4-point Likert scale ranging from 1=strongly agree to 4=strongly disagree. We abbreviated this survey to six items as follows: (1) “Vaccines are important for children to have, in general”; (2) “All childhood vaccines offered by my child’s health care provider are beneficial”; (3) “I do what my child’s health care provider recommends about vaccines”; (4) “Getting vaccines is a good way to protect my child from disease”; (5) “The information I receive about childhood vaccines from my child’s health care provider is reliable and trustworthy”; and (6) “Having my child vaccinated is important for the health of others in the community.” A principal components analysis was conducted to ensure these items were measuring a single domain, which yielded one factor that explained 78.2% of the overall variance. Higher scores on this measure indicate greater vaccine hesitancy.

#### Interviews

Open-ended, semistructured interviews were conducted by a lead interviewer and a notetaker who also ensured that all questions were asked and probed responses for more detail as necessary. Interviews were conducted via Zoom, telephone, or in-person based on participant preference and distance. The average duration for adult interviews was 91 minutes (range: 40-150 min), and youth interviews were 54 minutes (range: 36-77 min). Interview questions inquired about COVID-19 lived experiences at home, work, and school; vaccination status; views on current and future vaccines; coping and adaptations; resources needed; and public health policy and messaging recommendations. All interviewers received training on leading interviews. Training involved (1) practicing and debriefing to discuss modifications to improve the interview quality; (2) observing expert interviewers lead interviews with participants; and (3) leading interviews while expert interviewers took notes and provided feedback after each interview. Interviewers-in-training began leading interviews without expert interviewers present after both agreed they were ready. Interviews were transcribed verbatim, including nonverbal utterances.

### Data Analysis

For this protocol paper, data analyses are limited to descriptive statistics and psychometric properties of measures. These analyses were conducted using R (version 4.3.3; R Core Team) and SPSS Statistics (version 29.0; IBM Corp). Initially, we aimed to collect data from 900 participants within 1 year, as this would allow for more sophisticated modeling and subgroup analyses after accounting for missing data. After 1 year of data collection, we chose to stop data collection because the recall time period would be too long. The sample size of 599 was deemed sufficiently large for most of our analyses. For example, a sample size of 500 is widely accepted as standard practice for SEM models [47,48]. Further, post hoc power analyses were conducted using G*Power (version 3.1.9.7; Heinrich-Heine-Universitӓt Düsseldorf [49,50]) to evaluate the statistical power of regression models. For the adult sample (n=506), with 6 predictors, an α level of 0.05, and assuming a large effect size of *f*^2^=0.35, the achieved power (1-β) was >0.99. For the youth sample (n=93), using the same parameters, the achieved power was also >0.99.

Proposed studies will use quantitative analyses, including multiple regression and K-means or mixture modeling (latent profile analysis) to address the study aims. Group differences will be examined using ANOVA, followed by post hoc pairwise comparisons as appropriate. Qualitative data from open-ended survey responses and interviews will be analyzed using thematic analysis [51-53]. A consensus coding approach with independent coders will be used, using a multistage process: initial open coding, refinement into focused codes, axial coding, and final theme development via thematic analysis. Qualitative and mixed methods integration analyses will be conducted using MAXQDA (Version 2022; VERBI Software).

### Ethical Considerations

All procedures performed in studies involving human participants were in accordance with the ethical standards of the Institutional and National Research Committee and with the 1964 Helsinki Declaration and its later amendments or comparable ethical standards. The study was approved by the University of Illinois Urbana-Champaign Institutional Review Board (#24-0812). All data have been deidentified with unique numeric study IDs created for each participant. Digital copies of data are stored on university-maintained secure servers and password-protected computers. Hard copies of data are stored in locked file cabinets in a secure office. Pseudonyms will be used for qualitative data reporting. Audio and video files will be destroyed at the end of the study. Interested participants accessed a web-based eligibility screener with inclusion criteria questions. Participants meeting the criteria received a link to the web-based survey where they were asked to provide electronic consent via an information sheet prior to the start of the survey. The information sheet indicated that moving forward with the interview and answering questions represented their consent to the study and dissemination of findings via publications and presentations. Parents of children between the ages of 12 and 17 years could consent their children to participate in the study, and these children received a link to the main survey where they provided assent prior to the initiation of their web-based survey. Participants received an e-gift card compensation (US $20 for adults and US $15 for youth) after survey completion. Participants invited for the interviews received an additional US $25 e-gift card compensation.

## Results

### Overview

This study was funded in June 2022. Data collection concluded in August 2023 (except for one participant in March 2024), and data analysis, manuscript preparation, and dissemination are underway. To date, there are two published manuscripts using qualitative data from this study [13,18]. Regarding missing data, some instances arose from the diverse demographic characteristics of families and the survey’s branching logic, which rendered certain items inapplicable to specific participants. For example, measures of sibling-focused parentification were not applicable to adults without multiple children. In future papers, we plan to address missing data using multiple imputation or full information maximum likelihood, depending on the research questions.

### Participant Characteristics

Data were gathered from 506 adults, comprising 92.3% (467/506) female, 9.7% (49/506) Black, 4.7% (24/506) Hispanic, with 74.7% (378/506) reporting an annual household income of US $50,000 or more, and 16.8% (85/506) with incomes of US $49,000 or less. Additionally, 81.2% (411/506) were parents with children aged 17 years or younger, and 45.7% (231/506) held a graduate degree. The average age of adult participants was 42.8 (SD 9.15) years. The youth sample included 93 participants, 55.9% (52/93) of whom were female, 6.4% (6/93) Black, and 17.2% (16/93) Hispanic, with an average age of 14.4 (SD 1.63) years. Among the 506 adults, 166 were childcare providers, consisting of 98.2% (163/166) female, 16.9% (28/166) Black, and 3.6% (6/166) Hispanic. In this sample, there were 42 parent-child dyads, 3 parent-parent dyads, 2 parent-parent-child triads, and 21 parent-child-child triads. Demographic information for the adult and youth participants is summarized in Tables 3 and 4, respectively. The study flow from screening through study completion is presented in Figure 2.

**Table 3:** Demographic characteristics of adults (n=506).

Characteristic					Adults (n=506)	Interviewed adults (n=45)
Age (years), mean (SD)					42.8 (9.15)	43.8 (6.27)
Number of children, mean (SD)					2.1 (1.18)	2.2 (1.25)
**Number of children, n (%)**						
			Aged 17 years or younger		411 (81.2)	39 (86.7)
			Aged 18 years or older		111 (21.9)	15 (33.3)
			None		37 (7.3)	—^a^
			1 child		108 (21.3)	8 (17.8)
			2 children		214 (42.3)	19 (42.2)
			3 children		93 (18.4)	5 (11.1)
			4 or more children		49 (9.7)	9 (20)
			Missing		5 (1)	0 (0)
**Biological sex, n (%)**						
		Male			39 (7.7)	5 (11.1)
		Female			467 (92.3)	40 (88.9)
**Education, n (%)**						
		High school degree or GED^b^			23 (4.5)	—
		Some college but no degree			60 (11.9)	—
		Associate’s degree			42 (8.3)	—
		Bachelor's degree			150 (29.6)	19 (42.2)
		Graduate degree			231 (45.7)	21 (46.7)
**Employment, n (%)**						
			Unemployed		46 (9.1)	—
			Full-time		397 (78.5)	33 (73.34)
			Part-time		62 (12.2)	9 (20)
			Missing		1 (0.2)	0 (0)
**Income, n (%)**						
	US $50,000 or more				378 (74.7)	33 (73.3)
	US $49,000 or less				85 (16.8)	12 (26.7)
	Missing or prefer not to answer				43 (8.5)	0 (0)
**Marital status, n (%)**						
			Single or widowed		77 (15.2)	8 (17.8)
			Married or civil union		395 (78.1)	34 (75.6)
			Separated or divorced		34 (6.7)	—
**Ethnicity or Hispanic, n (%)**						
			Hispanic		24 (4.7)	—
			Non-Hispanic		482 (95.3)	41 (91.1)
**Race, n (%)**						
				American Indian or Alaska Native ^a^	—	—
				Asian or Native Hawaiian or Other PI ^a^	27 (5.3)	—
				Black	49 (9.7)	—
				White	402 (79.4)	36 (80)
		Multiracial			25 (4.9)	—
		Something else			—	—

^a^Indicate sample sizes of less than 5 participants.

^b^GED: General Education Development.

**Table 4:** Demographic characteristics of youth (n=93).

Characteristic			Youth (n=93)		Interviewed youth (n=21)
Age (years), mean (SD)			14.5 (1.63)		13.8 (1.31)
Missing, n			2		2
**Biological sex, n (%)**					
	Male	41 (44.1)		11 (52.4)	
	Female	52 (55.9)		10 (47.6)	
**Education, n (%)**					
	Sixth grade	7 (7.5)		—^a^	
	Seventh grade	18 (19.4)		5 (23.8)	
	Eighth grade	23 (24.7)		6 (28.6)	
	Nineth grade	16 (17.2)		—	
	10th grade	13 (14)		—	
	11th grade	11 (11.8)		—	
	12th grade	—		—	
**Ethnicity or Hispanic, n (%)**					
	Hispanic	16 (17.2)		5 (23.8)	
	Non-Hispanic	75 (80.6)		14 (66.7)	
	Missing	2 (2.2)		2 (9.5)	
**Race, n (%)**					
	Asian or Native Hawaiian or other PI^b^	—		—	
	Black	6 (6.4)		—	
	White	71 (76.3)		17 (81)	
	Multiracial	10 (10.8)		—	
	Something else	0 (0)		—	
	Missing	2 (2.2)		2 (9.5)	

^a^Indicate sample sizes of less than 5 participants.

^b^PI: Pacific Islander.

### Scale and Subscale Psychometric Properties

Internal consistency—an indicator of measurement reliability—was calculated for each scale and subscale (Cronbach α values are presented in Multimedia Appendix 1). A cutoff of 0.60 is considered an acceptable level of reliability for conducting analyses [53]. In this study, most scales and subscales met this threshold. For subscales with α values below 0.60, we explored item deletion to improve reliability values and found this approach increased values above 0.60 for the Parentification Inventory. The psychometric properties for each scale are presented in Multimedia Appendix 1.

## Discussion

### Principal Findings and Scientific Contribution

Using a mixed methods and multiple-informant approach, this study sheds light on the variety of lived experiences of parents, youth, families, and childcare providers during the COVID-19 pandemic. It will identify positive (silver linings) and negative (thorns) effects of the pandemic, successful and unsuccessful coping adaptations, and public health–relevant insights into how to prepare for future pandemics. These data will also enhance our understanding of behavioral and contextual factors influencing vaccine decision-making among families, youth, and childcare providers. At a general level, this knowledge will translate into how to tailor public health communication and health intervention campaign messaging to meet the differential needs of adults, youth, families, and childcare providers. At more specific levels, analysis of family functioning and dynamic, mental health, and attitudinal constructs will inform prevention and intervention recommendations (1) for families, youth, and childcare providers navigate the aftermath of the pandemic, as well as (2) for service providers and policy makers as they make decisions about resources (eg, infrastructure and programs) to prepare for future pandemics. Potential applications are detailed below.

This study will build upon current literature in several key ways. Our first aim—investigating how parental, childcare provider, and youth stress, mental health, and overload relate to individual coping and family functioning—allows us to examine shared and nonshared experiences over the course of the pandemic. Actor-partner models have examined dyadic reciprocity of social support on mental health outcomes at a single time point during the pandemic [4]. In this study, we ask questions across the course of the pandemic, which allows us to explore the dynamic temporal effects of the pandemic on outcomes such as household functioning, role reversal due to pandemic demands, including how other factors impacted this relationship, such as perceived economic hardships. These findings will inform how to adapt intervention and prevention programs to support the varied needs of family members rather than treating them as one-size-fits-all.

For childcare providers, we will explore how their experiences evolved over the course of the pandemic and how it impacted their home lives. Understanding these different needs will better inform prevention strategies and policies promoting resiliency and thriving during future public health crises.

COVID-19 detrimentally impacted people’s views on vaccines, partly because of misinformation, distrust in government agencies, conspiracies, fear of side effects, and inconsistent messaging [54]. Few studies have assessed the impact of COVID-19 on adolescents’ vaccine attitudes and beliefs to determine what factors contribute to vaccine behaviors. Rather, much of the literature focuses on parents’ attitudes about vaccinating their children. Additionally, this has not been investigated in family dyads and triads or in childcare providers. Our second aim—predicting vaccine attitudes and uptake patterns in youth, parents, and childcare providers—expands upon this literature to inform more tailored public health messaging.

There are two published qualitative studies using these data [13,18]. One of these studies highlights the importance of understanding how risk and reward are assessed by people to inform their decision-making [13]. For example, Dariotis et al [13] describe three case studies that had varied experiences with vaccines before and during the pandemic and had different vaccination behaviors (eg, early adopter, never adopter, and adopted but felt coerced). A major take-home message of the study was that all three participants valued protecting their loved ones and family, even though their choices differed from each other. This is very informative because the same public health messaging will not work for other people who have similar attitudes and beliefs as each of these cases. The other study highlights that the pandemic had both positive and negative outcomes for youth and families. For some youth, the pandemic facilitated accelerated maturity. For other youth, especially during early childhood, the pandemic had detrimental effects on speech and social skills. These are just a few examples of how these data contribute to the literature on pandemic-related experiences for multiple family members, across early life developmental stages through adolescence, and childcare settings.

### Strengths and Limitations

This study has numerous methodological strengths that address gaps in the literature. First, this study includes a large sample of parents who report on up to two children aged younger than 18 years, allowing for understanding parental perspectives on differential attitudes, behaviors, and impacts for multiple youth within families. Second, a subset of youth participants completed surveys and interviews, providing insights into shared and nonshared attitudes and experiences with parents and siblings. Taken together, having parents and youth in the same study allows for answering family systems research questions about the COVID-19 pandemic. Third, childcare providers are often invisible frontline workers, and hearing their perspectives is important for understanding pandemic impact and recommendations for future pandemics. In addition, studies of childcare providers typically examine the pandemic’s impact on their work and not their own families, a gap this study addresses. Fourth, the mixed methods nature of this study provides both larger-scale quantitative estimates for constructs of interest and rich, in-depth qualitative data that, when triangulated, will give a more holistic understanding of the pandemic, including thorns, silver linings, and lessons learned.

This study has limitations. First, some measures vary from the originally developed scales. This was primarily done to adapt to the pandemic and its context. Second, participants retrospectively report pre- and midpandemic attitudes and perceptions. The pandemic occurred so quickly that a prospective study was not possible. We acknowledge the potential for recall effects of retrospective accounts. We contend that these data have merit because beliefs about the pandemic—even if changed by memory—impact current and future behaviors and beliefs. Third, surveying and interviewing people with nonmainstream beliefs is a challenge given social desirability and stigmatizing pressures. We engaged in best practices to diversify our sample.

### Conclusions

In conclusion, with increasing distrust in government and health care communications and the potential for future public health crises, it is essential to comprehend the varied experiences of families, youth, and childcare providers during the COVID-19 pandemic. This encompasses both successful and unsuccessful adaptations, responses to policies and mandates, and unmet needs for health messaging, programs, policies, and services.

## Appendix

Multimedia Appendix 1 Psychometric properties of measures.

## Data Availability

These datasets generated or analyzed during this study are not publicly available due to privacy or ethical restrictions. The interview guides may be requested from the corresponding author.
