# Supplementary material for: COVID-19 Pandemic Experiences Among Adults, Youth, and Childcare Providers: Protocol for a Mixed Methods Study
Source: JMIR Res Protoc. 2025 Nov 28;14:e77521. doi: 10.2196/77521 (PMC12701342; doi:10.2196/77521)
Supplement: Multimedia Appendix 1 [file resprot_v14i1e77521_app1.docx]

| Scales | Number of Items | N | Alpha | Mean | SD | N | Alpha | Mean | SD |
| --- | --- | --- | --- | --- | --- | --- | --- | --- | --- |
| **Adults and Youth Report** |  | **Adult** | | | | **Youth** | | | |
| *Strengths and Difficulties Questionnaire (SDQ)* | 25 |  |  |  |  |  |  |  |  |
| **Pre** | | | | | | | | | |
| Hyperactivity | 5 | 229 | 0.71 | 3.7 | 2.5 | 82 | 0.75 | 4.7 | 2.4 |
| Emotional Symptoms | 5 | 229 | 0.80 | 1.9 | 2.1 | 82 | 0.47 | 3.7 | 1.9 |
| Conduct Problems^a^ | 5 | 229 | 0.57 | 1.4 | 1.5 | 82 | 0.60 | 2.0 | 1.8 |
| Peer Problems | 5 | 229 | 0.65 | 2.0 | 1.9 | 82 | 0.21 | 2.8 | 1.6 |
| Prosocial | 5 | 229 | 0.86 | 6.5 | 3.2 | 82 | 0.65 | 6.5 | 2.1 |
| Total Difficulties | 20 | 229 | 0.83 | 9.0 | 5.7 | 82 | 0.75 | 13.1 | 5.6 |
| *Confusion Hubbub and Order Scale (CHAOS)* | 15 |  |  |  |  |  |  |  |  |
| Pre |  | 482 | 0.75 | 29.4 | 6.6 | 84 | 0.79 | 3.4 | 7.0 |
| *Perceived Economic Hardship Questionnaire (PHQ)* | 17 |  |  |  |  |  |  |  |  |
| Not Enough Money for Necessities | 6 |  |  |  |  |  |  |  |  |
| Pre |  | 452 | 0.91 | 1.9 | 0.8 | NA | NA | NA | NA |
| Inability to Make Ends Meet | 2 |  |  |  |  |  |  |  |  |
|  |  | **Adult** | | | | **Youth** | | | |
| Pre |  | 452 | 0.78 | 2.7 | 0.4 | NA | NA | NA | NA |
| Financial Strain | 2 | 452 | 0.87 | 1.4 | 0 | 77 | 0.56 | 1.5 | 0.7 |
| Economic Adjustments | 7 | 381 | 0.94 | 2.2 | 1.1 | NA | NA | NA | NA |
| *Perceived Stress Scale (PSS)* | 10 | 481 | 0.90 | 16.0 | 7.0 | 80 | NA | 19.1 | 5.8 |
| *Personal Values (PV)* | 8 | 481 | 0.78 | 56.3 | 11.2 | 79 | NA | 54.7 | 13.1 |
| *Parentification Inventory (PI)* | 22 |  |  |  |  |  |  |  |  |
| Parent-Focused | 12 |  |  |  |  |  |  |  |  |
| Pre |  | 386 | 0.93 | 1.6 | 0.5 | 86 | 0.87 | 2.1 | 0.7 |
| Sibling-Focused^a^ | 7 |  |  |  |  |  |  |  |  |
| Pre |  | 391 | 0.80 | 2.1 | 0.7 | 86 | 0.64 | 2.7 | 0.7 |
| Perceived Benefits | 3 |  |  |  |  |  |  |  |  |
| Pre |  | 386 | 0.82 | 4.0 | 0.9 | 86 | 0.79 | 3.8 | 0.8 |
| *Media Attention (MA)* | 3 | 457 | 0.77 | 2.9 | 1.0 | 77 | 0.62 | 2.7 | 1.1 |
| *Extended Parallel Process Model (EPPM)* | 15 |  |  |  |  |  |  |  |  |
| Severity | 4 | 457 | 0.86 | 3.22 | 1.02 | 76 | 0.75 | 3.27 | .91 |
| Susceptibility | 5 | 457 | 0.76 | 3.12 | .76 | 76 | 0.60 | 2.79 | .73 |
| Mean Score | 9 | 457 | 0.87 | 3.17 | .80 | 76 | 0.77 | 3.00 | .71 |
| Response Efficacy | 6 | 457 | 0.94 | 3.6 | 1.2 | 76 | 0.91 | 3.86 | .89 |
| **Adults Report** | | | | | | | | | |
| *Depression Anxiety Stress Scale (DASS)* | 21 |  |  |  |  |  |  |  |  |
| Depression | 7 | 481 | 0.88 | 3.2 | 3.7 | NA | NA | NA | NA |
| Anxiety | 7 | 481 | 0.78 | 2.4 | 2.9 | NA | NA | NA | NA |
| Stress | 7 | 481 | 0.88 | 5.6 | 4.0 | NA | NA | NA | NA |
| *Psychological Reactance Scale (PRS)* | 14 |  |  |  |  |  |  |  |  |
| Freedom | 4 | 480 | 0.73 | 3.1 | 0.9 | NA | NA | NA | NA |
| Conformity Reactance | 3 | 480 | 0.74 | 2.0 | 0.8 | NA | NA | NA | NA |
| Behavioral Freedom | 4 | 480 | 0.68 | 2.5 | 0.8 | NA | NA | NA | NA |
| Reactance to Advice and Recommendations | 3 | 480 | 0.64 | 2.4 | 0.7 | NA | NA | NA | NA |
| *Conspiracy Mentality (CM)* | 5 | 457 | 0.79 | 3.4 | 0.8 | NA | NA | NA | NA |
| *Cultural Attitudes (CA)* | 12 |  |  |  |  |  |  |  |  |
| Hierarchy | 3 | 457 | 0.73 | 3.8 | 1.2 | NA | NA | NA | NA |
| Egalitarianism | 3 | 457 | 0.76 | 4.5 | 1.3 | NA | NA | NA | NA |
| Fatalism | 3 | 457 | 0.74 | 3.3 | 1.7 | NA | NA | NA | NA |
| Individualism | 3 | 457 | 0.83 | 3.2 | 1.3 | NA | NA | NA | NA |
| *Religiosity and Ideology (RI)* | 4 | 423 | 0.75 | 2.6 | 1.2 | NA | NA | NA | NA |
| *Parents Attitudes Toward Childhood Vaccines (PACV)* | 9 |  |  |  |  |  |  |  |  |
| Behavior | 3 |  |  |  |  |  |  |  |  |
| Pre |  | 469 | 0.69 | 3.5 | 0.9 | NA | NA | NA | NA |
| Safety and Efficacy | 3 |  |  |  |  |  |  |  |  |
| Pre |  | 469 | 0.87 | 2.0 | 0.8 | NA | NA | NA | NA |
| General Attitudes | 2 |  |  |  |  |  |  |  |  |
| Pre |  | 469 | 0.75 | 8.6 | 1.9 | NA | NA | NA | NA |
| Hesitancy | 1 |  |  |  |  |  |  |  |  |
| Pre |  | 469 | NA | 0.6 | 0.8 | NA | NA | NA | NA |
| *Vaccine Risk Analysis (VRA)* | 5 | 466 | 0.86 | 2.5 | 2.2 | NA | NA | NA | NA |
| *Vaccine Confidence Inventory (VCI)* | 6 |  |  |  |  |  |  |  |  |
| Covid Mean Score | 3 | 322 | 0.98 | 5.2 | 2.1 | NA | NA | NA | NA |
| Flu Mean Score | 3 | 322 | 0.98 | 5.6 | 1.7 | NA | NA | NA | NA |
| *Vaccine Hesitancy Scale (VHS)* | 5 |  |  |  |  |  |  |  |  |
| Pre |  | 465 | 0.88 | 3.3 | 0.5 | NA | NA | NA | NA |
| **Youth Report** | | | | | | | | | |
| PROMIS Measures (Patient-Reported Outcomes Measurement Information System) | | | | | | | | | |
| *Emotional-Behavioral Dyscontrol* | 8 | NA | NA | NA | NA | 76 | 0.91 | 21.1 | 6.5 |
| *Cognitive Functioning* | 8 | NA | NA | NA | NA | 76 | 0.83 | 27.9 | 5.8 |
| *Sleep-Related Disturbance* | 6 | NA | NA | NA | NA | 76 | 0.73 | 11.0 | 3.9 |
| *Emotional Distress-Anxiety* | 8 | NA | NA | NA | NA | 78 | 0.90 | 2.6 | 6.7 |
| *Depressive Symptoms* | 8 | NA | NA | NA | NA | 78 | 0.93 | 18.8 | 7.2 |
| *Positive Affect and Well-Being* | 9 | NA | NA | NA | NA | 78 | 0.90 | 32.6 | 6.5 |
| *Child Adolescent Mindfulness Measure (CAMM)* | 10 | NA | NA | NA | NA | 78 | 0.86 | 23.6 | 7.7 |
| *Emotion Regulation Questionnaire (ERQ)* | 10 |  |  |  |  |  |  |  |  |
| Cognitive Reappraisal | 6 | NA | NA | NA | NA | 78 | 0.80 | 4.4 | 1.0 |
| Emotion Suppression | 4 | NA | NA | NA | NA | 78 | 0.68 | 4.1 | 1.0 |
| *Brief COPE* | 28 |  |  |  |  |  |  |  |  |
| Self-Distraction | 2 | NA | NA | NA | NA | 76 | 0.55 | 3.2 | 1.5 |
| Instrumental Support | 2 | NA | NA | NA | NA | 76 | 0.77 | 2.6 | 1.6 |
| Active Coping | 2 | NA | NA | NA | NA | 76 | 0.55 | 2.5 | 1.4 |
| Behavioral Disengagement | 2 | NA | NA | NA | NA | 76 | 0.76 | 2.0 | 1.8 |
| Denial | 2 | NA | NA | NA | NA | 76 | 0.67 | 1.5 | 1.7 |
| Venting | 2 | NA | NA | NA | NA | 76 | 0.50 | 2.2 | 1.5 |
| Substance Use | 2 | NA | NA | NA | NA | 76 | 0.84 | 0.6 | 1.2 |
| Humor | 2 | NA | NA | NA | NA | 76 | 0.87 | 2.7 | 2.0 |
| Emotional Support | 2 | NA | NA | NA | NA | 76 | 0.66 | 2.6 | 1.7 |
| Positive Reframing | 2 | NA | NA | NA | NA | 76 | 0.64 | 2.7 | 1.6 |
| Planning | 2 | NA | NA | NA | NA | 76 | 0.68 | 2.8 | 1.5 |
| Acceptance | 2 | NA | NA | NA | NA | 76 | 0.63 | 3.1 | 1.6 |
| Religion | 2 | NA | NA | NA | NA | 76 | 0.90 | 2.3 | 2.0 |
| Self-Blame | 2 | NA | NA | NA | NA | 76 | 0.72 | 2.7 | 1.7 |
| *Adverse Childhood Experiences Checklist (ACEs)* | 8 | NA | NA | NA | NA | 71 | 0.64 | 0.38 | 0.85 |
| *Social Competence (SCS)* | 9 | NA | NA | NA | NA | 76 | 0.85 | 25.6 | 5.5 |

*^a^*To enhance the Alpha value, item 9 (“had time for play or school work even though he/she had family responsibilities”) from the Parentification Inventory (PI) was removed.
